# 62 More than a kickaround: A health impact assessment, over a twelve-month period, of Football Cooperative: A Men’s Community-based Social Initiative of Physical Activity

**DOI:** 10.1093/eurpub/ckae114.087

**Published:** 2024-09-26

**Authors:** Steve Daly, Paula Carroll, Tom Egan, Michael Harrison, Noel Richardson

**Affiliations:** Centre for Health Behaviour Research, South East Technological University, Waterford, Ireland; Centre for Health Behaviour Research, South East Technological University, Waterford, Ireland; Department of Accountancy & Economics, South East Technological University, Waterford, Ireland; Centre for Health Behaviour Research, South East Technological University, Waterford, Ireland; National Centre for Men’s Health, South East Technological University, Carlow, Ireland

## Abstract

**Purpose:**

It is well established that men suffer lower life expectancy and quality of life. Recognising the importance of gender-specific strategies in addressing men’s health, considerable evidence supports the notion that a substantial portion of male life expectancy and morbidity can be influenced by modifiable factors. Therefore, it is vital to create and expand impactful health interventions tailored for men, enhancing overall population health. This study evaluates the health impact of the participants in Football Cooperative (FC), a community-based physical activity and social initiative designed and used by men.

**Methods:**

Outcomes were measured for participants at four time points (baseline, three, six and twelve months) using both objective and subjective measures. Anthropometric measures included: a) height (cm), b) weight (Kg) (from which, body mass index (BMI) was calculated (Kg/m2)) and c) waist circumference (WC). Aerobic fitness was assessed using the Yo-Yo Intermittent Recovery test level 1. The self-reported data in the survey had a mix of demographic and psychological measures. These included, cardiovascular risk factors, UCLA loneliness scale and Rosenberg self-esteem scale.

**Results:**

With a mean age of 38 years (SD ± 7 years) n = 107 participants provided data. The average BMI went from 27.2 at baseline (B) to 26.2 at twelve months (12M). Average weight, decreased 87.4kg (B) to 84.3kg (12M) and WC decreased by 4.5%, from 97.1cm (B) to 92.7 (12M). In the self-report survey there were improvements in mental health, at three months (3M) 50% of participants reported improvements, increasing to 95% at 12M. Likewise, improvements in Social Health at 33% (3M) increased to 72% (12M). Participants reported decreased alcohol consumption and increased fruit and vegetable consumption. There was an increase in injuries sustained from 48% (3M) to 66% (12M). Stratifying by attendance, low attendees had less benefit than high (23% low to 67% high in physical benefit).

**Conclusion:**

The Football Cooperative initiative provided improvements in the health of the participants, which increased over time. It can be argued that engaging men in ongoing activity will benefit many health aspects over the lifetime.

**Funding Source:**

This PhD has been funded by the South East Research Development Fund [WD SERD_2020_54_WSCH, €90,000].

